# High Frequency of Resistance, Lack of Clinical Benefit, and Poor Outcomes in Capreomycin Treated South African Patients with Extensively Drug-Resistant Tuberculosis

**DOI:** 10.1371/journal.pone.0123655

**Published:** 2015-04-24

**Authors:** Elize Pietersen, Jonny Peter, Elizabeth Streicher, Frik Sirgel, Neesha Rockwood, Barbara Mastrapa, Julian Te Riele, Malika Davids, Paul van Helden, Robin Warren, Keertan Dheda

**Affiliations:** 1 Lung Infection and Immunity Unit, Division of Pulmonology and University of Cape Town Lung Institute, Department of Medicine, University of Cape Town, Cape Town, South Africa; 2 Department of Science and Technology/National Research Foundation Centre for Excellence for Biomedical Tuberculosis Research/South African Medical Research Council Centre for TB Research, Division of Molecular Biology and Human Genetics, Faculty of Medicine and Health Science, Stellenbosch University, Tygerberg, South Africa; 3 Gordonia Provincial Hospital, Upington, South Africa; 4 Department of Medicine, Imperial College London, London, United Kingdom; 5 Brooklyn Chest Hospital, Cape Town, South Africa; University of Delhi, INDIA

## Abstract

**Background:**

There are limited data about the epidemiology and treatment-related outcomes associated with capreomycin resistance in patients with XDR-TB. Capreomycin achieves high serum concentrations relative to MIC but whether capreomycin has therapeutic benefit despite microbiological resistance remains unclear.

**Methods:**

We reviewed the susceptibility profiles and outcomes associated with capreomycin usage in patients diagnosed with XDR-TB between August 2002 and October 2012 in two provinces of South Africa. Patients whose isolates were genotypically tested for capreomycin resistance were included in the analysis.

**Results:**

Of 178 XDR-TB patients 41% were HIV-infected. 87% (154/178) isolates contained a capreomycin resistance-conferring mutation [80% (143/178) *rrs* A1401G and 6% (11/178) were heteroresistant (containing both the *rrs* A1401G mutation and wild-type sequences)]. Previous MDR-TB treatment, prior usage of kanamycin, or strain type was not associated with capreomycin resistance. 92% (163/178) of XDR-TB patients were empirically treated with capreomycin. Capreomycin resistance decreased the odds of sputum culture conversion. In capreomycin sensitive and resistant persons combined weight at diagnosis was the only independent predictor for survival (p=<0.001). By contrast, HIV status and use of co-amoxicillin/clavulanic acid were independent predictors of mortality (p=<0.05). Capreomycin usage was not associated with survival or culture conversion when the analysis was restricted to those whose isolates were resistant to capreomycin.

**Conclusion:**

In South Africa the frequency of capreomycin conferring mutations was extremely high in XDR-TB isolates. In those with capreomycin resistance there appeared to be no therapeutic benefit of using capreomycin. These data inform susceptibility testing and the design of treatment regimens for XDR-TB in TB endemic settings.

## Introduction

Multi-drug resistant tuberculosis (MDR-TB) is a burgeoning problem worldwide with an estimated ~480 000 cases recorded globally in 2014 [1]. About 5–10% of cases of MDR-TB have extensively drug-resistant TB (XDR-TB) and some strains have evolved to resistance beyond XDR-TB (XXDR-TB or totally drug-resistant TB) [2–4]. Treating drug-resistant TB consumes almost 45% of the total budget of the South African National TB Programme (NTP) [5] and this scenario has the potential to destabilise successful TB treatment programmes in many high burden countries. Initial optimism about reasonably good outcomes [6, 7] have been supplanted by more dismal data from high burden setting(s) [8–12], indicating a high mortality and culture conversion rates of less than 20%. The factors underpinning the poor outcomes in high burden settings compared to intermediate burden settings, are not well understood.

Patients with XDR-TB are resistant to four potent anti-TB drugs (rifampicin, isoniazid, fluoroquinolones and aminoglycosides) and in South Africa, resistance to the latter two drugs is mostly acquired (i.e. a high proportion of cases have been infected with a circulating MDR-TB strain). This in part is due to a weakened MDR-TB regimen because of the unrecognized high level of ethionamide resistance [13]. Given that alternative drugs like linezolid are not available to resource poor national TB programmes, therapeutic options are severely limited, and capreomycin forms the backbone of a presumed effective empiric regimen. Although capreomycin has been used since 2006 in South Africa, capreomycin susceptibility testing only became more widely available after 2010 and thus the overall levels of resistance to this drug, despite empiric use, has been poorly studied [14, 15]. Given the above-mentioned considerations we reasoned that capreomycin resistance might be significant, be associated with prior aminoglycoside usage, and may explain the poor treatment outcomes [16, 17]. Furthermore, given that peak serum levels attained with capreomycin are well above the minimum inhibitory concentration (MIC) [18, 19], we hypothesised that capreomycin could still have a therapeutic benefit despite the presence of the *rrs* A1401G mutation conferring *in vitro* resistance, according to the WHO defined critical concentration (2.5ug/ml) in MGIT media [20]. By contrast, lack of benefit is also likely to inform patient management as we recently showed that capreomycin is a toxic drug with significant morbidity and mortality [21], and an expensive drug that may be inappropriately diverting resources away from effectively functioning segments of the NTP [5]. Thus, defining the context-specific risk-benefit ratio of capreomycin is critical. Such data also inform advocacy efforts to accelerate the development of new anti-TB drugs and trial of immunotherapeutic options in patients with XDR-TB.

To address these unanswered questions, we reviewed the susceptibility profiles, associated risk factors, and treatment outcomes of patients with XDR-TB in whom bio-banked isolates were available for *rrs* genotyping.

## Materials and Methods

### Setting and patients

We retrospectively reviewed the case records of 310 patients (>18 years) with culture proven XDR-TB diagnosed between August, 2002 and October 2012 at two of nine dedicated provincial facilities for the treatment of XDR-TB in South Africa. Data including regimens, treatment start and stop dates, adverse-events, and treatment outcomes were recorded

### Definitions and diagnosis of MDR-TB, Pre-XDR TB and XDR-TB

Pre-XDR TB is defined as resistance to rifampicin, isoniazid and either a fluoroquinolone or a second line injectable drug (amikacin, kanamycin or capreomycin). Standard definitions for MDR-TB and XDR-TB are outlined in the online supplement (S1.1 in S1 Definitions and Methods).

### Outcomes

Early treatment outcomes were sputum culture conversion and reversion and late treatment outcomes were treatment cure/completion, death, default, treatment failure or transfer out. Death was the primary outcome measure in this study. Culture conversion was defined as two consecutive negative sputum cultures at least 30 days apart. Culture reversion was defined as two consecutive positive sputum cultures at least 30 days apart after initial sputum culture conversion. Death was all-cause mortality, not necessarily secondary to TB progression.

### Drug susceptibility testing

Isolates underwent routine phenotypic drug susceptibility testing (DST) (rifampicin, isoniazid, ofloxacin, amikacin and ethionamide) in the centralised NTP-designated reference laboratory (NHLS) as previously described [22]. Drug susceptibility testing to terizidone, fluroquinolones other than ofloxacin and para-aminosalicylic acid is unavailable within the provincial laboratories. Targeted DNA sequencing of the *inhA* promoter and the *katG*, *rpoB*, *embB*, *pncA*, *gyrA*, and *rrs* genes was used to identify mutations conferring resistance [2]. Based on data from a previous study about the frequency of mutations conferring phenotypic resistance to capreomycin in the *rrs* gene (A1401G, G1484T) and *tylA* gene in clinical MDR-TB, Pre-XDR TB and XDR-TB isolates from the Eastern Cape, South Africa, the *rrs* A1401G mutation was selected to identify genotypic resistance to capreomycin [15]. Thus, isolates harbouring this mutation, including heteroresistance (presence of both the *rrs* A1401G mutation and wild-type *rrs* sequences), were designated as resistant. Targeted DNA sequencing of the *rrs* gene was used to identify the *rrs* A1401G mutation [15] (See online supplement (S1.2 in S1 Definitions and Methods) for full details). DST of the clinical isolates against capreomycin was carried out according to the standard proportion method on Middlebrook 7H10-agar as suggested by the National Committee on Clinical Laboratory Standards at a critical concentration of 10.0 μg/ml [23]. Stock solutions of the drug were prepared in distilled water and sterilised by filtration through a Millex-GV 108 syringe-driven filter with a membrane pore size of 0.22 μm. Aliquots of stock solutions were then stored at -80°C in screw-cap polypropylene cryovials up to 6 months. Further dilutions were made in sterile distilled water as required.

MIC testing against capreomycin was done using the MGIT 960 system with EpiCenter TB eXiST software on a subset of isolates which were susceptible to capreomycin according to the standard proportion method. A MIC equal to the critical concentration was reported as susceptible, while MICs ≥2.5 μg/ml [15] was considered resistant [18, 20]. More detailed methods are outlined in the online supplement (S1.3 in S1 Definitions and Methods).

### Molecular epidemiology

A subset of 126 isolates from patients from the Western Cape Province was genotyped by use of spoligotyping [22].

### Statistical analysis

We compared categorical variables by use of the χ^2^ or Fisher exact test where appropriate, and we compared continuous variables using the Mann-Whitney U or Kruskal-Wallis tests. Univariate and multivariate logistic regression analysis was used to control for confounding and identify associates of capreomycin genotypic resistance, XDR-TB mortality and sputum culture conversion. See online supplement (S1.4 in S1 Definitions and Methods) for further details.

### Ethics Statement

Ethical approval was obtained from the University of Cape Town human research ethics committee. Patient information was anonymised and de-identified prior to analysis.

## Results

### Study cohort and demographic data

**Fig 1:**
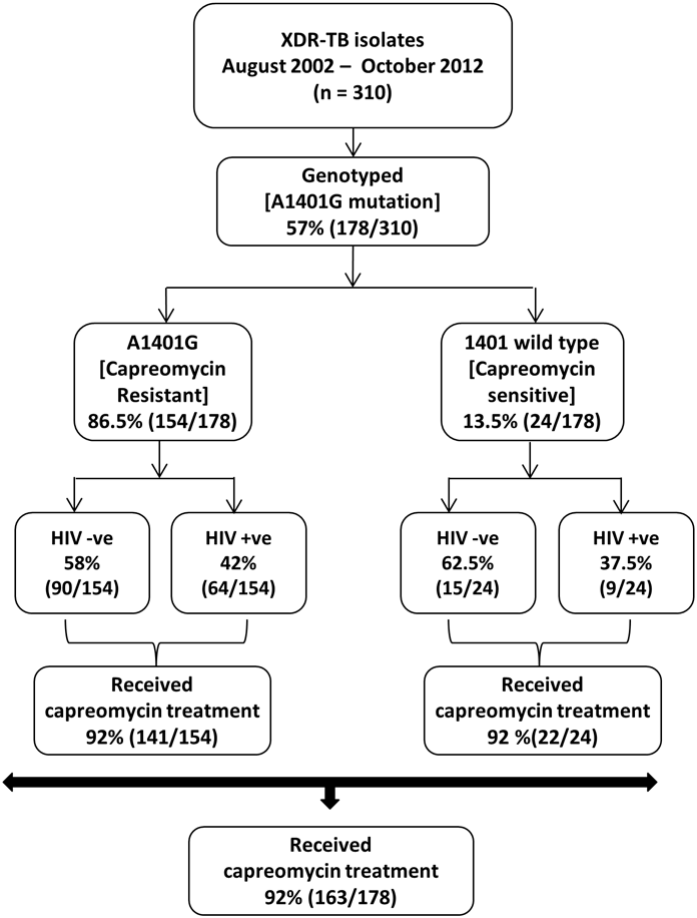
Study plan showing the relationship between capreomycin genotypic susceptibility profile (wild type, *rrs* A1401G mutation), HIV status, and proportion of participants who received capreomycin.

Between August 2002 and October 2012 57% (178/310) of the XDR-TB patients who were commenced on treatment were genotyped for the *rrs* A1401G mutation. No differences in the age, gender, ethnicity and HIV serostatus was noted between genotyped included and non-genotyped excluded XDR-TB patients (data not shown). The median age of genotyped patients was 33 years (IQR 27–41 years), 55% were male, 47% were of mixed ancestry and the median weight at diagnosis was 50.4kg (Table 1). 41% of the cohort was HIV-infected with a median CD4 count of 193 (IQR 99–379 cells/mm^3^) and 87% were receiving antiretroviral therapy (ART) at XDR-TB diagnosis. Of the 126 isolates spoligotyped, 83% were the Beijing genotype (Table 1). 60% had a previous diagnosis of MDR-TB and 13% were Pre-XDR TB. 67% of the cohort had previous exposure to either amikacin or kanamycin. The median total number of drugs in the regimen was 8 (IQR 7–9) and 92% of regimens were inclusive of capreomycin (Table 1). Capreomycin resistance as per the presence of the *rrs* A1401G mutation was present in the isolates from 87% (154/178) of patients who were predominantly HIV-uninfected [58% (90/154)] (Fig 1). The demographic profile and clinical characteristics were not significantly different when comparing isolates from patients with *rrs* A1404G mutation compared to wild type (Table 1).

**Table 1:** Demographic profile, clinical characteristics and treatment related outcomes in patients with XDR-TB stratified by *rrs* A1401G mutation status.

Patient characteristic	All	*rrs* A1401G mutation	*rrs wild type*	P-value
	(N = 178) n (%)	(N = 154) n (%)	(N = 24) n (%)	
**Demographics**				
Age, yrs, median (IQR)	33 (27–41)	33 (26–41)	31 (28–40)	n/s
Male	97 (55)	85 (55)	12 (50)	n/s
Mixed ancestry	84 (47)	72 (47)	12 (50)	n/s
HIV-infected	73 (41)	64 (42)	9 (38)	n/s
CD4 count, cells/μl, median (IQR) #	193 (99–379)	193 (99–365)	213 (112–486)	n/s
Receiving anti-retroviral therapy at diagnosis	61/70 (87)	52/61 (85)	9/9 (100)	n/s
Weight at diagnosis, kgs, median (IQR)	50.4	50.3	50.9	n/s
	(44.4–60)	(44.4–61.1)	(44–57.9)	
**TB history—sputum culture proven**				
Previous MDR-TB	103 (60)	85 (57)	18 (75)	n/s
Previous Pre-XDR TB^†^	23 (13)	21 (14)	2(8)	n/s
**TB strain**				
Beijing genotype strain	105/126 (83)	93/109 (85)	12/17 (71)	n/s
**Treatment exposure—ever**				
SLID^§^	119 (67)	101 (68)	18 (75)	n/s
Capreomycin	163 (92)	141 (92)	22 (92)	n/s
Ofloxacin	110 (62)	97 (63)	13 (54)	n/s
Moxifloxacin	29 (16)	26 (17)	3 (13)	n/s
Number of drugs in regimen, median (IQR)	8 (7–9)	8 (7–9)	8 (7–9)	n/s
**XDR-TB treatment outcomes** ¶				
Converted	53 (31)	42 (28)	11 (42)	n/s
Reverted	18/53 (34)	16/42 (38)	2/11 (19)	n/s
Mortality	93 (53)	78 (52)	15 (63)	n/s

^#^4/73 HIV-infected patients missing CD4 cell count data and 3/73 patients missing ARV data.

^†^Previous Pre-XDR TB is defined as MDR-TB plus resistance to either FQ or second line injectable drugs.

^§^SLID: Second-line injectable drug (either kanamycin or amikacin).

^¶^4/178 and 3/178 missing accurate conversion and mortality data respectively.

Abbreviations: IQR: interquartile range, n/s: not significant (p = >0.05), n/c: not calculated.

### Capreomycin susceptibility data

We compared capreomycin phenotypic (via agar proportions method) and genotypic susceptibility for 51% (91/178) of isolates (Table 2). There was concordance for capreomycin susceptibility in 79% (72/91) of isolates. 16 of the 19 discordant isolates were available for further analysis and discordance was consolidated using the MGIT 960 system (MIC shown in Table 3). 100% of these isolates, excluding three with contaminated or non-viable MGIT 960 cultures, were reclassified as resistant (Table 3). Resistance in the 3 isolates found to be phenotypically resistant with no *rrs* A1401G mutation present could potentially be explained by mutations outside the *rrs* region (unknown mechanisms conferring resistance) or an underlying population not detected by DNA sequencing.

**Table 2:** Comparison of capreomycin phenotypic and genotypic drug susceptibility.

Phenotypic DST^ζ^	Capreomycin genotype		Total
	*rrs* A1401G	*rrs* wild type	
**Sensitive**	16^†^	10	26
**Resistant**	62	3*	65
**Total**	78	13	91

^ζ^Phenotypic DST on Middlebrooks 7H10-agar was performed using the current critical concentration for capreomycin of 10μg/ml (1). Phenotypic results were only available for 91/178 patients with *rrs* genotyping results.

^†^16/19 discordant isolates that were available were re-tested using MGIT (see Table 3).

*3/19 discordant isolates may be due to mutations outside the *rrs* region or an underlying population not detected by DNA sequencing.

**Table 3:** Minimum inhibitory concentrations (MIC) using the MGIT 960 phenotypic drug-susceptibility method for initially genotypic resistant, phenotypic sensitive (agar-based) discordant capreomycin isolates.

*rrs* genotype	Initial phenotypic classification using ECOFF for capreomycin (10μg/ml) on 7H10 agar	MGIT 960 MIC (μg/ml)	Phenotypic reclassification
A1401G	S	10	R
A1401G/A	S	5	R
A1401G	S	>10	R
A1401G	S	5	R
A1401G	S	Contaminated	n/r
A1401G	S	5	R
A1401G	S	10	R
A1401G	S	5	R
A1401G	S	5	R
A1401G	S	5	R
A1401G	S	5	R
A1401G	S	10	R
A1401G	S	5	R
A1401G	S	Contaminated	n/r
A1401G	S	5	R

MGIT: Microscopic growth in-tube (BD Biosciences, USA); S: Sensitive, R: Resistant, n/r: no result

### Relationship between capreomycin susceptibility and treatment-related outcomes

The overall cohort mortality was 53% (93/175) with three patients missing data, culture conversion was 31% (53/174) with four patients missing data, and culture reversion was 34% (18/53 patients that converted). 92% (141/154) of isolates with the *rrs* A1401G mutation received capreomycin as part of their XDR-TB regimen while 92% (22/24) of patients with wild type *rrs* isolates received capreomycin (Fig 1). When not stratified by capreomycin treatment status, there were no significant differences in the above-mentioned short-term clinical outcomes between those with an isolate with an *rrs* A1404G mutation versus those with the wild type *rrs* genotype. (Table 1) However, in an analysis uncorrected for potential confounders, the mortality was significantly higher in patients (resistant and sensitive capreomycin genotype combined) that did not receive capreomycin treatment compared to those that did [86% (12/14) versus 50% (81/161), p = 0.01]. Restricted to patients with genotypic resistance mortality was also higher in patients who did not receive capreomycin compared with those who did [83% (10/12) versus 49% (68/139) p = 0.022] (Table 4; there were 2 patients with missing data).

**Table 4:** Mortality, sputum culture conversion and sputum culture reversion in XDR-TB patients classified by capreomycin genotype and treatment status.

Capreomycin genotype	Mortality	Sputum culture conversion	Sputum culture reversion
	(n/N, %)	(n/N, %)	(n/N, %)
**Capreomycin treatment given (N = 163)**			
*Resistant and sensitive combined (n = 163)*	81/161 (50.3)* ^1^	50/163 (30.7)	17/50 (34.0)
*rrs* A1401G wild type (sensitive) (n = 22)	13/22 (59.1)	10/22 (45.5)	1/10 (10.0)
*rrs* A1401G (resistant) (n = 141)	68/139 (48.9)* ^2^	40/141 (28.4)	16/40 (40.0)
**No capreomycin treatment given (N = 15)**			
*Resistant and sensitive combined (n = 15)*	12/14 (85.7)* ^1^	3/11 (27.3)	1/3 (33.3)
	*1p = 0.011		
*rrs* A1401G wild type (sensitive) (n = 2)	2/2 (100.0)	1/2 (50.0)	1/1 (100.0)
*rrs* A1401G (resistant) (n = 13)	10/12 (83.3)* ^2^	2/9 (22.2)	0/2 (0)
	* ^2^p = 0.022		

*p-values are for χ^2^ testing between proportions for different capreomycin treatment status but similar genotypic DST results. Only significant p-values shown (p = <0.05).

^1^ mortality in all patients (resistant and sensitive) treated and not treated with capreomycin

^2^ mortality only in patients with *rrs* A1404G mutation treated and not treated with capreomycin

The following numbers of patients were missing mortality outcome data: i) 2/141 of isolates from patients with *rrs* A1401G mutations receiving capreomycin treatment, and ii) 1/13 isolates from patients with *rrs* A1401G mutations not receiving capreomycin treatment. 4/13 isolates from patients with *rrs* A1401G mutations not receiving capreomycin treatment are missing sputum conversion data.

Sputum culture conversion and reversion was not significantly different in any of the capreomycin-specific susceptibility categories irrespective of capreomycin treatment (Table 4).

### Impact of strain type and HIV co-infection on capreomycin resistance and treatment outcomes

Strain type by spoligotyping and HIV infection were not associated with capreomycin genotypic resistance (Table 1). Mortality in HIV-infected patients was significantly higher than in HIV-uninfected patients [62% (44/71) versus 47% (49/104) p = 0.05]. Mortality in HIV-infected patients whose isolates were capreomycin resistant was not significantly different when compared to isolates from HIV-uninfected patients. Sputum culture conversion did not vary significantly, when stratified by HIV sero-status and when comparing HIV-infected patients whose isolates were capreomycin resistant versus those whose isolates were capreomycin sensitive.

### Multivariate analysis and factors associated with outcomes

#### Mortality

In a multivariate analysis looking at predictors of mortality in the genotyped study population, only weight at diagnosis [OR 0.935 (95% CI 0.902–0.969), p = <0.001] and HIV infection [OR 2.9 (95% CI 1.3–6.3), p = 0.007] was associated with decreased and increased odds of mortality, respectively. Neither capreomycin genotype resistance (p = 0.32) nor capreomycin treatment (p = 0.16) had a significant impact controlling for confounders. Similar significant predictors of mortality were noted when restricting the multivariate analysis to i) only patients receiving capreomycin treatment and ii) only patients with capreomycin resistance, with the addition of co-amoxicillin/clavulanic acid treatment as a significant predictor of mortality in both sub-group analyses (Table 5).

**Table 5:** Multivariate logistic regression analysis for predicators^†^ of XDR-TB mortality in the study cohort with resistance to *rrs* A1401G.

	*rrs* A1401G mutation present versus *rrs* wild type	
Variable	Odds ratio (95% CI)	P-value
**All XDR-TB patients (n = 158/178)** *		
Weight at diagnosis (kgs)	0.935 (0.902–0.969)	<0.001
HIV-infected	2.9 (1.34–6.3)	0.007
Capreomycin *rrs* resistance (A1401G mutation	0.59 (0.21–1.65)	0.32
TB drugs impacting mortality		
Capreomycin	0.27 (0.04–1.67)	0.16
Moxifloxacin	0.39 (0.14–1.05)	0.06
Co-amoxicillin/clavulanic acid	3.1 (1.4–6.6)	0.004
**Only XDR-TB patients treated with capreomycin (n = 151/163)** *		
Weight at diagnosis (kgs)	0.94 (0.91–0.98)	0.001
HIV-infected	2.7 (1.2–5.9)	0.01
Capreomycin *rrs* resistance (A1401G mutation	0.64 (0.22–1.83)	0.4
TB drugs impacting mortality		
Moxifloxacin	0.4 (0.15–1.11)	0.08
Co-amoxicillin/clavulanic acid	3.2 (1.4–7.2)	0.004
**Capreomycin resistant (defined by *rrs* A1401G mutation) n = 136/154)** *		
Weight at diagnosis (kgs)	0.950 (0.917–0.984)	0.005
HIV-infected	2.6 (1.2–5.8)	0.02
TB drugs impacting mortality		
Capreomycin	0.28 (0.04–1.87)	0.19
Moxifloxacin	0.49 (0.18–1.32)	0.16
Co-amoxicillin/clavulanic acid	3.3 (1.5–7.1)	0.003
**Only *rrs* wild type (capromycin sensitive) (n = 21/24)** *		
Weight at diagnosis (kgs)	0.758 (0.589–0.976)	0.03
HIV-infected	22.3 (0.29–1698.2)	0.16
TB drugs impacting mortality		
Moxifloxacin	0.01 (0.00–12.7)	0.2
Co-amoxicillin/clavulanic acid	6.9 (0.26–181.5)	0.25

^†^A priori variables included in both the univariate and mulitivariate analysis included i) Demographic and clinical: Age, gender, ethnicity, weight at diagnosis, HIV status, CD4 cell count and ART (in HIV-infected), previous MDR-TB treatment, previous pre-XDR diagnosis; ii) *M*. *tuberculosis* strain typing and drug-susceptibility testing: Beijing/non-Beijing strain, phenotypic DST for second line injectables (amikacin, kanamycin, capreomycin, streptomycin), ofloxacin, ethambutol, ethionamide, and capreomycin genotypying for *rrs* A1401G mutant, iii) XDR-TB drug treatments: amikacin, kanamycin, capreomycin, ciprofloxacin, ofloxacin, moxifloxacin, co-amoxicillin/clavulanic, ethambutol, ethionamide, pyrazinamide, PAS, clofazime, dapsone, thioacetone, terizidone/cycloserine.

*The total numbers of observations included in each of the multivariate outputs. Numbers differ from previous patient group totals due to missing data. Multiple imputation was not used.

#### Culture conversion

In the genotyped study population weight at diagnosis [OR 1.063 (95% CI 1.027–1.101), p = 0.001] was associated with an increased odds of sputum culture conversion. By contrast, capreomycin resistance [OR 0.64 (95% CI 0.11–0.68), p = 0.007] and previous MDR-TB treatment [OR 0.45 (0.21–0.97), p = 0.04] were associated with decreased odds of sputum culture conversion. Capreomycin in the regimen and usage of ≥8 drugs was not significantly associated with culture conversion. Restricting the analysis to only those receiving capreomycin treatment, weight at diagnosis [OR 1.059 (95% CI 1.023–1.097), p = 0.001] was associated with an increased odds of sputum culture conversion; Capreomycin resistance [OR 0.28 (95% CI 0.10–0.78), p = 0.02] was associated with a decreased odds of culture conversion (Table 6).

**Table 6:** Multivariate logistic regression analysis for predicators^†^ of XDR-TB sputum culture conversion in the study cohort with resistance to *rrs* A1401G.

	*rrs* A1401G mutation present versus *rrs* wild type	
Variable	Odds ratio (95% CI)	P-value
**All XDR patients (n = 160/178)** *		
Weight at diagnosis (kgs)	1.063 (1.027–1.101)	0.001
Previous MDR-TB treatment	0.45 (0.21–0.97)	0.04
HIV-infected	0.93 (0.43–2.01)	0.86
Capreomycin *rrs* resitance	0.64 (0.11–0.68)	0.007
Capreomycin	0.64 (0.12–3.50)	0.6
**Only XDR patients treated with capreomycin (n = 153/163)** *		
Weight at diagnosis (kgs)	1.059 (1.023–1.097)	0.001
Previous MDR-TB treatment	0.48 (0.22–1.03)	0.06
HIV-infected	0.92 (0.42–2.01)	0.84
Capreomycin *rrs* resitance	0.28 (0.10–0.78)	0.02

^†^A priori variables included in both the univariate and mulitivariate analysis included i) Demographic and clinical: Age, gender, ethnicity, weight at diagnosis, HIV status, CD4 cell count and ART (in HIV-infected), previous MDR-TB treatment, previous pre-XDR diagnosis; ii) *M*. *tuberculosis* strain typing and drug-susceptibility testing: Beijing/non-Beijing strain, phenotypic DST for second line injectables (amikacin, kanamycin, capreomycin, streptomycin), ofloxacin, ethambutol, ethionamide, and capreomycin genotypying for *rrs* A1401G mutant, iii) XDR-TB drug treatments: amikacin, kanamycin, capreomycin, ciprofloxacin, ofloxacin, moxifloxacin, co-amoxicillin/clavulanic, ethambutol, ethionamide, pyrazinamide, PAS, clofazime, dapsone, thioacetone, terizidone/cycloserine.

*The total numbers of observations included in each of the multivariate outputs. Numbers differ from previous patient group totals due to missing data. Multiple imputation was not used.

On multivariate analysis, strain type, CD4 count and use of ART (the latter 2 co-variates included in HIV-infected individuals only) did not impact the outcomes of mortality and culture conversion.

## Discussion

There are currently limited data on the frequency and factors associated with capreomycin resistance in high burden settings. The key findings of our study were (i) a high rate (87%) of capreomycin resistance in capreomycin-naive patients with XDR-TB despite the drug not previously being used in this population, (ii) capreomycin usage in patients whose isolates were resistant to the drug produced no detectable clinical benefit (conversion or mortality); rather, capreomycin resistance was a marker of conversion failure, (iii) capreomycin resistance was not associated with prior aminoglycoside usage, and (iv) despite an intensive in-patient multi-drug regimen treatment-related outcomes of patients with XDR-TB were poor.

In the cohort as a whole capreomycin use appeared to have a survival benefit despite genotypic resistance. However, after correcting for potential confounders, including HIV status, moxifloxacin and co-amoxicillin/clavulanic usage and previous MDR-TB, capreomycin usage was not associated with beneficial treatment-related outcomes (mortality and culture conversion) both overall and in those that were capreomycin resistant. We have recently shown that capreomycin toxicity is associated with serious morbidity and mortality in patients with XDR-TB [21]. All the drug-related adverse-event deaths were due to capreomycin (renal failure) and capreomycin made up more than half of all the drugs withdrawn [21]. Our data suggest, given the substantial costs of the drug and attendant toxicity, that capreomycin DST be routinely implemented in the NTP and those resistant to capreomycin should not be given the drug. Given that capreomycin is universally used in XDR-TB regimens in South Africa, this will also enable the channelling of substantial resources to more effective drugs like linezolid. Cost awareness is critical as we recently showed that DR-TB, despite comprising <3% of the caseload, already consumes almost 45% of the NTP budget which is not sustainable [5].

In patients with capreomycin sensitive TB, our study was not powered to make a definitive conclusion about mortality or culture conversion, as sample numbers were small. A retrospective study in a European population found that capreomycin was favourably associated with outcomes in patients with XDR-TB [17]. Although capreomycin is likely to have benefit in such patients, impact may be limited in the African setting as there are few additional effective drugs to which isolates are susceptible. Isolates in our study were already resistant to rifampicin, isoniazid, aminoglycosides and fluoroquinolones, and given the rate of previous MDR-TB, most were also resistant to ethionamide and terizidone (a compound of cycloserine). Thus, even in patients with capreomycin-sensitive TB it is likely that a single potentially effective drug would have limited impact on successful outcomes in patients with extensive lung disease and few other effective drugs to which the isolate is susceptible (linezolid is not an option as this is not available to the national TB programme).

It is unclear why the de novo capreomycin resistance rates in this capreomycin naive population were so high. Capreomycin is known to be associated with aminoglycoside resistance given that mutations conferring resistance to both classes of drugs are encoded by the *rrs* gene [14]. However, capreomycin resistance in this cohort seemed independent of aminoglycoside usage (25% of the cohort did not receive prior aminoglycosides). Thus, it is possible that transmission of capreomycin-resistant strains led to their amplification at the community level in the Western and Northern Cape provinces. We think this hypothesis is tenable given that a high proportion of patients who had no prior aminoglycoside usage also had high rates of capreomycin resistance and that person-to-person spread is the primary modality of transmission in MDR-TB with aminoglycoside resistance (80% of MDR-TB in South Africa is now due to primary transmission) [13].

Our findings on capreomycin susceptibility and associated outcomes were independent of HIV-status though this may represent a type 2 error. Degree of host immunosuppression in HIV-infected patients, as measured by CD4 count, did not appear to be an important risk factor for capreomycin resistance or treatment related outcomes. Similarly, the strain type did not impact results.

There are several limitations to our findings, including those inherent in a study with a retrospective design. There is a selection bias as those without capreomycin drug susceptibility data were excluded from the analysis. However, a sensitivity analysis showed that the excluded population had similar characteristics to those included in the study. A second major limitation is that there were only small numbers of patients who were capreomycin-sensitive and received the drug. Thus, we were underpowered to directly answer this question and we can make limited deductions about how effective the drug is in sensitive versus resistant patients (given survival benefit in the cohort as a whole). Although this is speculative, we feel that, even in capreomycin-sensitive patients, only one active drug is unlikely to have a sustained impact in patients who have extensive disease and where there are no other effective therapeutic options (although these patients also receive drugs like co-amoxicillin/clavulanic acid, clarithromycin and clofazimine however, these are of dubious value).

In conclusion, in this retrospective study we found that there was a high rate of capreomycin resistance, even in a population where there was no prior usage of capreomycin, and also in those who had not received prior aminoglycosides. Further, capreomycin usage in those resistant to capreomycin did not produce any beneficial therapeutic effect. Collectively, these data suggest that rapid genotypic testing (e.g Genotype®MTBD*sl* that is available) should be routinely made available to the South African NTP, and that capreomycin, given its costs and toxicity profile, should not be used for perceived therapeutic benefit in patients resistant to the drug (in contradistinction to current practice). Additional studies in other high burden settings are needed to confirm our findings. These data have important implications for the optimal design of drug regimes in high burden settings, and suggest that capreomycin should not be used in patients who have genotypic resistance to this drug. This may have important implications for resource allocation and costs borne by national TB programmes in high burden settings. These data also intensify the urgency with which new anti-TB drugs need to be developed.

## Supporting Information

S1 Dataset (XLSX)

S1 Definitions and Methods High frequency of resistance, lack of clinical benefit, and poor outcomes in capreomycin treated South African patients with extensively drug-resistant tuberculosis. (DOCX)

## References

[1] WHO. Global Tuberculosis Report 2014. Available: http://apps.who.int/iris/bitstream/10665/137095/1/WHO_HQ_TB_2014.12_eng.pdf?ua=1 17.12.14)

[2] Klopper M, Warren RM, Hayes C, Gey van Pittius NC, Streicher EM, Muller B, et al Emergence and spread of extensively and totally drug-resistant tuberculosis, South Africa. Emerg Infect Dis 2013; 19: 449–455. 10.3201/EID1903.120246 23622714 PMC3647643

[3] Migliori GB, Sotgiu G, Gandhi NR, Falzon D, Deriemer K, Centis R, et al Drug resistance beyond XDR-TB: results from a large individual patient data meta-analysis. Eur Respir J 2013; 42: 169–179. 10.1183/09031936.00136312 23060633 PMC4498806

[4] Udwadia ZF, Amale RA, Ajbani KK, Rodrigues C. Totally drug-resistant tuberculosis in India. Clin Infect Dis 2012; 54: 579–581. 10.1093/cid/cir889 22190562

[5] Pooran A, Pieterson E, Davids M, Theron G, Dheda K. What is the cost of diagnosis and management of drug resistant tuberculosis in South Africa? PLoS One 2013; 8: e54587 10.1371/journal.pone.0054587 23349933 PMC3548831

[6] Keshavjee S, Gelmanova IY, Farmer PE, Mishustin SP, Strelis AK, Andreev YG, et al Treatment of extensively drug-resistant tuberculosis in Tomsk, Russia: a retrospective cohort study. Lancet, 2008; 372: 1403–1409. 10.1016/S0140-6736(08)61204-0 18723218

[7] Mitnick CD, Shin SS, Seung KJ, Rich ML, Atwood SS, Furin JJ, et al Comprehensive treatment of extensively drug-resistant tuberculosis. New Engl J Med 2008; 359: 563–574. 10.1056/NEJMoa0800106 18687637 PMC2673722

[8] Dheda K, Shean K, Zumla A, Badri M, Streicher EM, Page-Shipp L, et al Early treatment outcomes and HIV status of patients with extensively drug-resistant tuberculosis in South Africa: a retrospective cohort study. Lancet 2010; 375: 1798–1807. 10.1016/S0140-6736(10)60492-8 20488525

[9] Kvasnovsky CL, Cegielsky JP, Erasmus R, Siwisa NO, Thomas K, van der Walt ML. Extensively drug-resistant TB in Eastern Cape, South Africa: high mortality in HIV-negative and HIV-positive patients. J Acquir Immune Defic Syndr, 2011; 57: 146–152. 10.1097/QAI.0b013e31821190a3 21297482

[10] O'Donnell MR, Jarand J, Loveday M, Padayatchi N, Zelnick J, Werner L, et al High incidence of hospital admissions with multidrug-resistant and extensively drug-resistant tuberculosis among South African health care workers. Ann Intern Med 2010; 153: 516–522. 10.7326/0003-4819-153-8-201010190-00008 20956708 PMC3074259

[11] O'Donnell MR, Padayatchi N, Master I, Osburn G, Horsburgh CR. Improved early results for patients with extensively drug-resistant tuberculosis and HIV in South Africa. Int J Tuberc Lung Dis 2009; 13: 855–861. 19555535 PMC2855970

[12] Pietersen E, Ignatius E, Streicher EM, Mastrapa B, Padanilam X, Pooran A, et al Long-term outcomes of patients with extensively drug-resistant tuberculosis in South Africa: a cohort study. Lancet 2014; 383: 1230–1239. 10.1016/S0140-6736(13)62675-6 24439237

[13] Streicher EM, Muller B, Chihota V, Mlambo C, Tait M, Pillay M, et al Emergence and treatment of multidrug resistant (MDR) and extensively drug-resistant (XDR) tuberculosis in South Africa. Infection, genetics and evolution 2012; 12: 686–694. 10.1016/j.meegid.2011.07.019 21839855

[14] Georghiou SB, Magana M, Garfein RS, Catanzaro DG, Catanzaro A, Rodwell TC. Evaluation of genetic mutations associated with Mycobacterium tuberculosis resistance to amikacin, kanamycin and capreomycin: a systematic review. PLoS One 2012; 7: e33275 10.1371/journal.pone.0033275 22479378 PMC3315572

[15] Sirgel FA, Tait M, Warren RM, Streicher EM, Bottger EC, van Helden PD, et al Mutations in the rrs A1401G gene and phenotypic resistance to amikacin and capreomycin in Mycobacterium tuberculosis. Microb Drug Resist 2012; 18: 193–197. 10.1089/mdr.2011.0063 21732736

[16] Jacobson KR, Theron D, Victor TC, Streicher EM, Warren RM, Murray MB. Treatment outcomes of isoniazid-resistant tuberculosis patients, Western Cape Province, South Africa. Clin Infect Dis 2011; 53: 369–372. 10.1093/cid/cir406 21810750 PMC3202325

[17] Migliori GB, Lange C, Centis R, Sotgiu G, Mutterlein R, Hoffmann H, et al Resistance to second-line injectables and treatment outcomes in multidrug-resistant and extensively drug-resistant tuberculosis cases. Eur Respir J 2008; 31: 1155–1159. 10.1183/09031936.00028708 18515555

[18] Annonymous. Capreomycin. Tuberculosis (Edinb) 2008; 88: 89–91. 10.1016/S1472-9792(08)70004-0 18486038

[19] Peloquin CA. Pharmacology of the antimycobacterial drugs. The Medical clinics of North America 1993; 77: 1253–1262. 8231410 10.1016/s0025-7125(16)30191-2

[20] WHO. Policy guidance on drug-susceptibility testing (DST) of second-line antituberculosis drugs. Geneva: WHO; 2008. 26290924

[21] Shean K, Streicher E, Pieterson E, Symons G, van Zyl Smit R, Theron G, et al Drug-associated adverse events and their relationship with outcomes in patients receiving treatment for extensively drug-resistant tuberculosis in South Africa. PLoS One 2013; 8: e63057 10.1371/journal.pone.0063057 23667572 PMC3646906

[22] Streicher EM, Bergval I, Dheda K, Bottger EC, Gey van Pittius NC, Bosman M, et al Mycobacterium tuberculosis population structure determines the outcome of genetics-based second-line drug resistance testing. Antimicrob Agents Chemother 2012; 56: 2420–2427. 10.1128/AAC.05905-11 22330913 PMC3346650

[23] Park YK, Koh WJ, Kim SO, Shin S, Kim BJ, Cho SN, Lee SM, et al National Committee for Clinical Laboratory Standards. Susceptibility testing of mycobacteria, norcardiae and other aerobic actinomycetes. Approved Standards. NCCLS document M24-A 2003;2023(2018):2001:2071.

