# Supplementary material for: High Frequency of Resistance, Lack of Clinical Benefit, and Poor Outcomes in Capreomycin Treated South African Patients with Extensively Drug-Resistant Tuberculosis
Source: PLoS One. 2015 Apr 24;10(4):e0123655. doi: 10.1371/journal.pone.0123655 (PMC4409172; doi:10.1371/journal.pone.0123655)
Supplement: S1 Definitions and Methods — (DOCX) [file pone.0123655.s002.docx]

**S1 - ONLINE SUPPLEMENT: Definitions and methods**

**High frequency of resistance, lack of clinical benefit, and poor outcomes in capreomycin treated South African patients with extensively drug-resistant tuberculosis.**

Elize Pietersen^1^, Jonny Peter^1^, Elizabeth Streicher^2^, Frik Sirgel^2^, Neesha Rockwood^4^, Barbara Mastrapa^3^, Julian Te Riele^5^, Malika Davids^1^, Paul van Helden^2^, Robin Warren^2^, Keertan Dheda^1^

^1^ Lung Infection and Immunity Unit, Division of Pulmonology and University of Cape Town Lung Institute, Department of Medicine, University of Cape Town, South Africa.

^2^Department of Science and Technology/National Research Foundation Centre for Excellence for Biomedical Tuberculosis Research/South African Medical Research Council Centre for TB Research, Division of Molecular Biology and Human Genetics, Faculty of Medicine and Health Science, Stellenbosch University, Tygerberg, South Africa.

^3^ Gordonia Provincial Hospital, Upington, South Africa.

^4^ Department of Medicine, Imperial College London, United Kingdom.

^5^ Brooklyn Chest Hospital, Cape Town, South Africa.

Corresponding author: ^†^Corresponding author: Keertan Dheda, Division of Pulmonology, Depertment of Medicine, H47 Old Main Building, Groote Schuur Hospital, Observatory, 7925, South Africa. Tel.: +27214046509; Fax: +27214047651; Email: keertan.dheda@uct.ac.za.

**S1.1 Definitions and diagnosis of MDR-TB, Pre-XDR TB and XDR-TB**

MDR-TB is defined at resistance to rifampicin and isoniazid. Pre-XDR TB is defined as resistance to rifampicin, isoniazid and either a fluoroquinolone or a second line injectable drug (amikacin, kanamycin or capreomycin). In this study patients with isolates of *M. tuberculosis* that were resistant at diagnosis (time of sputum collection) to at least isoniazid, rifampicin, a fluoroquinolone and at least one of the second-line injectable drugs (amikacin drug susceptibility testing was performed in almost all patients; capreomycin susceptibility was only available in a minority of patients) were judged to have extensively drug-resistant tuberculosis (XDR-TB).

S1.2 Genotyping

DNA sequencing of the region encompassing nucleotide 1401 of the *rrs* gene (amplification product nucleotide 1339 to 1528). Briefly, a 200 µl aliquot of the MGIT culture was heat inactivated by incubating at 100°C for 30 min to generate a crude DNA lysate. PCR amplification was done in a reaction mixture containing 2 µl crude DNA template, 5 µl Q-Buffer, 2.5 µl 10 x Buffer, 2 µl 25 mM MgCl2, 4 µl 10 mM dNTPs, 1 µl of the *rrs* primer set (Forward 5'-GTAATCGCAGATCAGCAAC-3' and Reverse 5'- GTGATCCAGCCGCACCTT -3'), 0.125 µl HotStarTaq DNA polymerase (Qiagen, Germany) and made up to 25 µl with dH2O. Amplification was initiated by incubation at 95°C for 15 minutes, followed by 35 - 45 cycles at 94°C for 45 seconds, 62°C for 45 seconds, and 72°C for 45 seconds. After the last cycle, the samples were incubated at 72°C for 10 minutes. To minimize laboratory cross-contamination the preparation of the PCR reaction mixes, the addition of the DNA and the PCR amplification were conducted in physically separated rooms. Negative controls (water) were included to detect reagent contamination. Amplification was confirmed by electrophoretic fractionation in 1% agarose containing TBE pH 8.3. Amplification products were sequenced using the ABI3130XL genetic analyzer and the resulting chromatograms were analysed using Chromas software

**S1.3 Capreomycin MICs**

MIC testing against capreomycin was done using the MGIT 960 system with EpiCenter TB eXiST software on a subset of isolates which were susceptible to capreomycin according to the standard proportion method. Briefly, the isolates were tested against serial twofold dilutions of capreomycin ranging from 0.125 to 10 µg/ml. *M. tuberculosis* strain H37Rv (ATCC 27294) was included as a capreomycin susceptible control. The MIC was defined as the lowest concentration of drug that inhibited more than 99% of the bacterial population, relative to the 1:100 diluted drug-free controls of the corresponding strains (1% proportional method). A critical concentration of 2.5 µg/ml was adopted to differentiate between capreomycin susceptible and resistant strains as suggested in previous publications (1). A MIC equal to the critical concentration was reported as susceptible, while MICs ≥2.5 µg/ml was considered resistant (2, 3).

**S1.4 Statistical analysis**

A risk management strategy for data, including double data entry, was used to ensure the integrity of the data. Descriptive statistics were used for the demographic and clinical characteristics of the study population. The genotyped study population was compared with overall XDR-TB cohort to ensure that the sample was representative. We compared categorical variables by use of the χ^2^ or Fisher exact test where appropriate, and we compared continuous variables, because of the non-normal distribution of the analysed variables, using the Mann-Whitney U or Kruskal-Wallis tests. Univariate and multivariate logistic regression analysis was used to control for confounding and identify associates of capreomycin genotypic resistance, XDR-TB mortality and sputum culture conversion. All statistical tests were 2-sided at α=0.05. STATA IC, version 11 (Stata Corp, Texas, USA) was used for all statistical analyses.
